# First Days in Africa

**Published:** 1887-09-24

**Authors:** 


### FIRST DAYS IN AFRICA.

By a Medical Missionary.

The mail leaves to-morrow morning at 8 o'clock, and it is now 9 o'clock p.m., so you must be content with something less than a detailed history of the past six weeks.

To begin with, we are at B——, in good health and spirits. I forget where I wrote from last—I think it was somewhere up the Shire; at any rate, one thing I do remember is that in my last letter I promised to give you a detailed account of our adventures in my next, a promise which was made in order to be broken. The journey up the river was very enjoyable in spite of very many "sticks"; the new steamer is much too big for the river, and the result was that almost every day we stuck on one or more sandbanks, and sometimes for a long time; one day we only made about five miles altogether. The scenery almost the whole way up is really fine, not so fine as Loch Lomond or the Clyde, but still worthy of being called fine; the immediate banks of the river have nothing particular about them, but some little distance inland the country rises gradually into finely-wooded hills, which are continued on for many miles.

As regards adventures we had none; certainly we saw a good number of crocodiles and "hippos," but we were in as much danger from crocodiles in the English Channel as on board the river steamer. One day when we were out in a small boat visiting Bishop Mackenzie's grave, we got a fright from a bull hippo, who stood in the water at a corner waiting for us; but F—— M——, who was with us, shot it, and it tore away badly wounded. We also saw two herds of elephants at a very short distance; we fired at them, but did not succeed in killing any.

On the 17th June about four p.m. we reached K——, the end of our river journey; from K—— to B—— it is twenty-eight miles up hill. On the 18th, the anniversary of our wedding day, we started at half-past nine in the morning, S——, B——, Miss B——, and Mrs. T—— in wachillas, the rest of us on our feet. I may say it was my first walk in Africa, and certainly it was the hardest day's walk I ever had in my life; before I had gone three miles up the hill with the African sun shining I was fairly wading through perspiration; the wonder is that anything but my clothes reached B——. After going six miles we stopped for half-an-hour and had some tea; then on till we reached half-way house; there we stopped about two hours and had something to eat; the something was not very much, as we had eaten the steamer out of provisions; we had rice without any salt, two small tins of soup also saltless, one tin of sausages, which we had to heat in the tin, no biscuits, no bread, some tea, and a few hard-boiled eggs,—very meagre fare, especially for us, as we had not been able to get any breakfast, there being literally nothing on board to eat. About half-past three we started with fifteen miles to walk, the longest fifteen miles I have gone over; however, even African miles have an end, and about eight in the evening, when we reached B——, both D—— and I were about as tired as men could be; I could hardly crawl along.

Of course you will be very anxious to hear what we new comers think of that marvellous place B——. Not an easy task to describe the place; certainly none of the descriptions I have read of it at all describe it as it is. B—— is amongst the hills, and yet is itself flat; it is on a level plateau surrounded by seven or eight good-sized hills, but the hills are some distance away, the nearest big one being five or six miles distant. The view of these from B—— is very fine, recalling very vividly the hills in the Western Highlands of Scotland. The hills are fairly well wooded, but with trees not tropically luxuriant in their foliage, and the natives cut down so many that the slopes and summits have a somewhat poorly clad appearance; then towards the top of the hills they become rocky, and the grey-faced rocks give a cold and northern homelike appearance; indeed, landed at B——, and looking at the hills around, one would say you were on the Scotch mountains.

The station, B—— itself, is very like what it is, a small settlement in a far country, with many home plants introduced, and many home comforts, certainly a very flourishing settlement, and one containing members with excellent taste. I don't know what to liken it to at home; it is a sort of cross between the entrance to a gentleman's house, a small flower nursery, and a pretty cemetery without any tombstones. After passing up a long avenue of blue gum-trees—a fairly pretty avenue, a very fine one for Africa, but not strikingly beautiful for home, the blue gums not being a very ornamental tree—you enter B—— proper; at its entrance you have houses on both sides, separated by a plot of about forty yards width, finely laid out into a central walk and borders of flowers. This continues for about fifty yards, and constitutes the famous square; then the square opens out into a wider and more mixed part; to the right, at some little distance behind the level of the square, are the school and native huts; to the left, ending the left side of the square, is the church, a small mud erection, by no means striking in appearance. Continuing onwards by the church you come to two large plots with lemon-trees, orange-trees, etc. Between these plots the road to the manse passes, the latter being about fifty yards from the church and square. The manse is about twenty yards in from the road, and the plot in front is beautifully laid out with home flowers, any number of geraniums, nasturtiums, stocks, etc., etc.

I must stop describing, as I really have no time to continue it. B—— is a very pretty place—pretty describes it; it is not grand, it is not lovely; the immediate station is naturally a flat, uninteresting piece of ground, but is now made fine by care and cultivation. It is very civilised in appearance. It is not like a beautiful little spot amongst the hills at home, with here a lovely little dell and there a little hill, with babbling burns going cheerily on their way; the beauty B—— has it owes to the mission. This applies to B—— only, not to the surroundings; they are much finer.

About a week after reaching here I had my first attack of fever; was in bed for six days with a tolerably bad attack; it is not a pleasant trouble,—a horrid pain in the back, head like to split, and bad vomiting; however, I am all right now.

How do I like B——? Am I disappointed with it? One cannot help liking it, and although the place is not at all what I expected, I am not disappointed; indeed, in time I shall like it much better than if it were as tropical and remarkable as I anticipated it would be. At first it is disappointing, because it is very like home, with nothing strange or striking about it, and yet it is not like home, because you miss the variety of home. We have not yet had any letters from England, but are expecting them every day. Be sure you write at least every month, and give any amount of gossip. I hope you are remembering to send (beginning with July) the German medical periodical. Dr. M—— is away to Z——, so I am in charge here. The foundation of our house is laid, and we hope to be in it by the new year at the latest.

Further details and descriptions I shall give you in some future letter.

